# Colored Proteins Act as Biocolorants in *Escherichia coli*

**DOI:** 10.3390/molecules30030432

**Published:** 2025-01-21

**Authors:** Geng Sun, Chunmei Zha, Jingwen Su, Feng Cheng, Jian Tang, Xiuquan Xu, Jincai Li, Wenjian Wang, Yu Liu

**Affiliations:** 1 School of Chinese Medicine, Bozhou University, Bozhou 236800, China; sungeng_sg@outlook.com (G.S.);; 2 Key Laboratory of Chinese Medicine Materials Research of Anhui Higher Education Institutes, Bozhou 236800, China

**Keywords:** GfasPurple, colored proteins, biocolorants, protein-only chromoproteins

## Abstract

Colored proteins play an important role in synthetic biology research, providing a systematic labeling tool for visualizing microscopic biological activities in vivo. They can exhibit visible colors to the naked eye under natural light, and some of them are well-known fluorescent proteins. Here, several colored proteins were taken into consideration for acting as biocolorants in *Escherichia coli*, including green fluorescent proteins (eGFP and sfGFP), a red fluorescent protein (mKate2), and three chromoproteins (GfasPurple, AmilCP, and AeBlue). All of them can significantly change the colors of their bacterial colonies. The color of GfasPurple was much more stable after the heat treatments at 65 °C with 75% or 95% ethanol. In addition, several factors commonly occurring under natural conditions that lead to color dissolution, such as heat, ethanol, H_2_O_2_, vitamin C, acid, and alkali treatments, were further tested on GfasPurple. Visual observation and absorption spectroscopy analysis results showed an excellent tolerance of GfasPurple against these unfriendly conditions. GfasPurple could withstand temperatures of 65 °C for 2 h or 70 °C for 1 h in aqueous solutions, but it fades rapidly in 50% ethanol. The color of GfasPurple is more stable in 80% ethanol than in 50% ethanol, which could be attributed to its poor solubility in high-concentration ethanol. The visible light absorption curves of GfasPurple were basically not affected by physiological concentrations of vitamin C or H_2_O_2_, but reversible effects of high-concentration H_2_O_2_ were found. GfasPurple maintains its color within the pH range of 7–11; the chromophore of GfasPurple will suffer irreversible damage when pH is up to thirteen or as low as three. These results suggest that GfasPurple is an excellent biocolorant far beyond its application in prokaryotes. Furthermore, GfasPurple variants created via mutagenesis expanded the color library of chromoproteins, which have a potential value in the color manipulation of living organisms.

## 1. Introduction

Colored proteins are widely present in various organisms in nature. Most of them have prosthetic groups with different colors, such as hemoglobin, cytochrome, chlorophyll, phycocyanin, and rhodopsin, which contain iron porphyrins, pyrrole derivatives, or retinal [1,2,3]. Colored proteins can work as molecular labels or colorants in scientific research, medical diagnosis, and even in food and chemical industries, and they have the great potential to be utilized as synthetic targets in synthetic biology. For example, phycocyanin is a rare natural blue pigment. In European Union countries, phycocyanin is a food additive that can be used with unlimited quantities as food colorants [4]. In addition, some of them are protein-only biocolorants without non-protein cofactors, such as green fluorescent protein, which is made of 238 amino acids; their chromophore consists only of its amino acid residues [5]. Specifically, fluorescent proteins are a class of fluoresce chromoproteins that have been widely studied and applied in the life science field, and they are used as easily imaged molecular markers [6,7].

Protein-only chromoproteins (POCPs), non-fluorescent or fluorescent, have a better potential application value than those with pigment cogroup. POCPs have vividly visible pigmentation under natural light due to the chromophores made from their amino acid residues, which are hidden in their β-barrel structure [8]. The formation of their cofactor-free chromophore only requires oxygen, making their application very simple compared to those with pigment cogroups. In addition, the monomers of POCPs encoded by monogenes facilitate their cloning and engineering for imaging in vivo.

POCPs often work as dimers, tetramers, or even monomers [9,10,11]. Their monomers often have similar β-barrel structures in the secondary structure despite the significant differences they may have in homology. To date, dozens of POCPs have been discovered and researched, especially those with fluorescence [12]. The research and application of non-fluorescent POPCs appear relatively inadequate compared to those with fluorescence. Interestingly, the vast majority of POCPs originate from marine invertebrates, such as jellyfish, coral polyps, and sea anemones [6].

Here, several POCPs, colored yellow, red, and blue, under natural light were tested in *E. coli* to identify more stable POCPs, with the expectation that they act as biocolorants. Hence, non-fluorescent or fluorescent POCPs were taken into consideration. Both enhanced green fluorescent protein (eGFP) and super-folder GFP (sfGFP) appear yellow to the naked eyes [13,14]. MKate2 is a rapidly maturing red fluorescent protein monomer published in 2009; it was derived from *Entacmaea quadricolor*, and it displays as red [15]. AeBlue is a non-fluorescent POCP normally displayed as blue, and it was derived from *Actinia equina*, and it was reported as a temperature-dependent color [16]. GfasPurple is a violet protein derived from *Galaxea fascicularis* first published in 2008 [17]. AmilCP is a similar bluish-violet protein derived from *Acropora millepora*; it is a homologous protein with only four amino acid differences against GfasPurple [8,17]. These eukaryotes-derived colored proteins were successfully expressed in *E. coli* strain DH5α using a very simple strategy.

## 2. Results

### 2.1. Production, Extraction, and Spectral Analysis of Colored Proteins

All the POCP genes were well expressed in DH5α on solid or liquid LB medium via the pSG-CBSA001 backbone described above, except AmilCP which hardly displayed blue after transformation, while the others could easily display their colors as expected. Although AmilCP only shares 4 amino acid differences against GfasPurple, they have different expressive characteristics. DH5α harbors an AmilCP expression cassette barely colored in the first five days after transformation, but it could display a pale blue color at day 3 when using the bacteria cryopreserved at −80 °C. This phenomenon has not been found in other cases.

**Figure 1:**
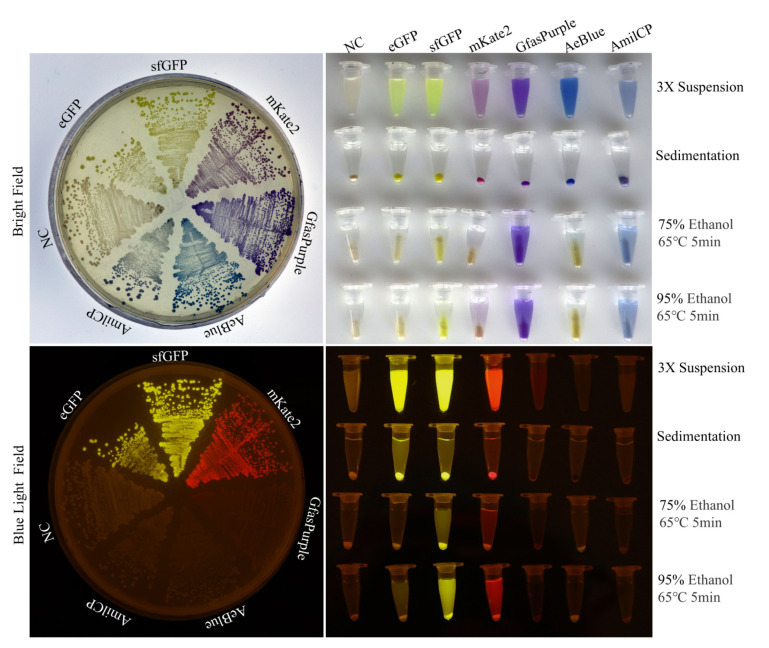
Expression and heating test with ethanol of POCPs in *E. coli* in 3X suspension: triple enrichment of bacterial culture products.; Sedimentation: equivalent bacterial pellets as in 3X suspension; NC: negative control with the empty vector backbone; resuspended in water to avoid the influence of LB medium color after treatments.

POCPs expressed in solid or liquid LB medium showed consistent colors and fluorescence properties (Figure 1). The yellow colors of eGFP and sfGFP on the plate were not obvious because of the background color of the LB medium. mKate2 appeared purple and displayed red under blue excitation light. GfasPurple and AeBlue appeared purple and blue under white light, and no obvious fluorescence phenomenon was found under blue excitation light.

In the process of searching for high-temperature and ethanol-resistant chromoproteins, a heating experiment conducted at 65 °C was conducted with the presence of 75% or 95% ethanol. The results showed that sfGFP was more tolerant to the ethanol and heat treatment than eGFP. These treatments resulted in less impact on GfasPurple and AmilCP, but they caused AeBlue to display weak fluorescence (Figure 1). Similar phenomena can be found in separated heat treatments at 70 °C, 80 °C, and 90 °C, as shown in Figure S2A. While the negative control harbored no chromoproteins, it displayed a slight natural grayish yellow after the relatively long cultivation time. An overall trend observed was that these POCPs were more stable in 95% ethanol than in 75% ethanol; the GfasPurple and AmilCP were more stable under these conditions than the others, while GfasPurple even displayed color with better absorption capacity. Thus, GfasPurple became a key focus of the subsequent research.

**Figure 2:**
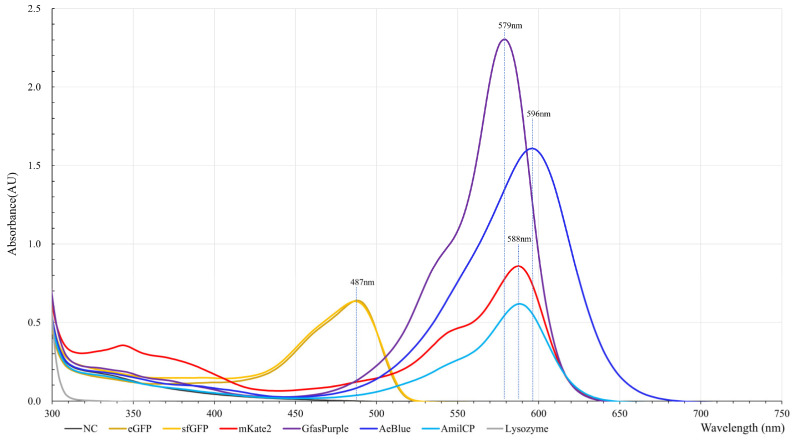
Absorption spectroscopies of crude protein extraction solutions. NC: negative control with an empty vector backbone.

The absorption spectroscopies analysis revealed that eGFP and sfGFP have a consistent absorption of yellow light at around 487 nm, and this makes the display yellow since it is the complementary color of yellow. GfasPurple has an absorption peak at 579 nm with a less conspicuous shoulder peak at around 530 nm, which makes it purple (Figure 2). AmilCP and mKate2 have consistent absorption peaks at 588 nm, while mKate2 has a distinct shoulder peak at around 545 nm. This shoulder peak and the red emission light of mKate2 make it red, unlike AmilCP which displays as bluish violet. Aeblue has a wide absorption peak of around 596 nm, which matches the absorption of orange light and thus makes it deep blue.

### 2.2. Heat and Ethanol Tolerance Capabilities of GfasPurple

**Figure 3:**
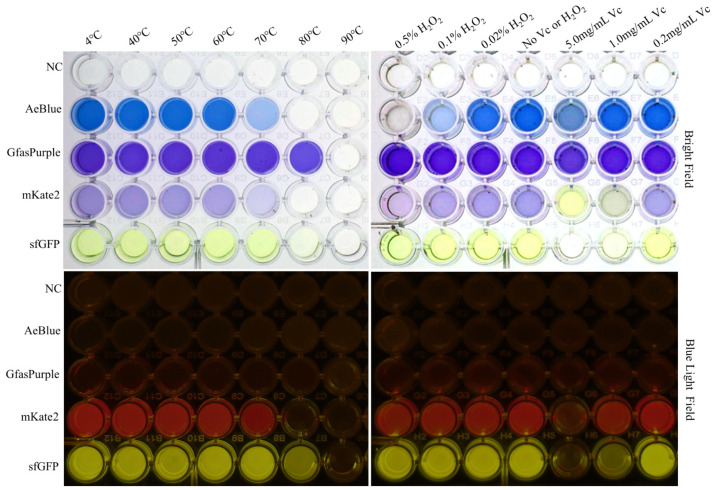
Tolerance tests against heat, H_2_O_2_, and vitamin C. All processing times are 60 min with the reaction temperature at 37 °C unless stated otherwise. Vc: vitamin C.

To further explore the effect of temperature on these proteins, a temperature gradient test (40–90 °C, step = 10 °C) was conducted on sfGFP, mKate2, GfasPurple, AeBlue, and the negative control, which contains an uncolored extract of chromoprotein-less *E. coli*. None of them withstood the test at 90 °C for 60 min, and all of them eventually turned into a white sediment (Figure 3). The samples in the microplate were also simultaneously detected at their respective absorption peaks using a microplate reader with a multi-wavelength measurement system. Only GfasPurple withstood temperatures at 70 °C for up to 60 min without an obvious loss of color. Even under 80 °C, GfasPurple had a loss of no more than 20%. As a comparison, sfGFP reduced by over 80%, while AeBlue and mKate2 loss all of their colors (Figure S1).

**Figure 4:**
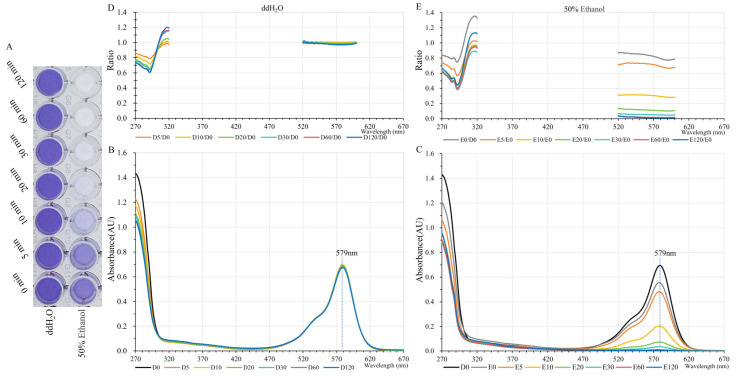
Heat and ethanol tolerance tests of GfasPurple at 65 °C. (**A**) Heat treatments of GfasPurple in water or 50% Ethanol. Absorption spectroscopies of heated GfasPurple in water (**B**) and 50% ethanol (**C**). The relative ratio curves of the absorption value versus D0 in water (**D**) or 50% ethanol (**E**) at 270–320 nm and 520–600 nm.

An endurance time test was also conducted with or without the presence of 50% ethanol. GfasPurple persisted for 2 h without fading at 65 °C in an aqueous solution, while it quickly faded and barely expressed color in 50% ethanol for 20 min (Figure 4A). A subsequent absorption spectroscopy analysis confirmed that GfasPurple is very stable in water at 65 °C, with the light absorption curves at different time points almost completely overlapped in the visible light range. In the near-ultraviolet region, absorption curves move downwards over time (Figure 4B). This phenomenon indicates that the lysozyme and host proteins separate from the crude extraction solution and produce insoluble precipitation in prolonged high-temperature treatments. When 50% ethanol is present, absorption curves move downwards over time at around the 579 nm peak, with a more significant decrease in the near-ultraviolet region than in water (Figure 4C).

The relative ratio in treated versus untreated samples (D0 in Figure 4B) was calculated and plotted at 270–320 nm and 520–600 nm where the absorbance value is appreciable. The approximate horizontal curves at 520–600 nm indicate that the two absorption curves are similar (Figure 4D,E). In other words, the light absorption properties of GfasPurple have not changed, only the amount of GfasPurple in the solution has changed after the heat treatments, both in water and in 50% ethanol (Figure 4B,C). The trend of relative ratio curves at 270–320 nm significantly differed, indicating that the proportion of protein loss is distinct between the water treatment and the 50% ethanol treatment.

### 2.3. H_2_O_2_ and Vitamin C Tolerance Capabilities of GfasPurple

**Figure 5:**
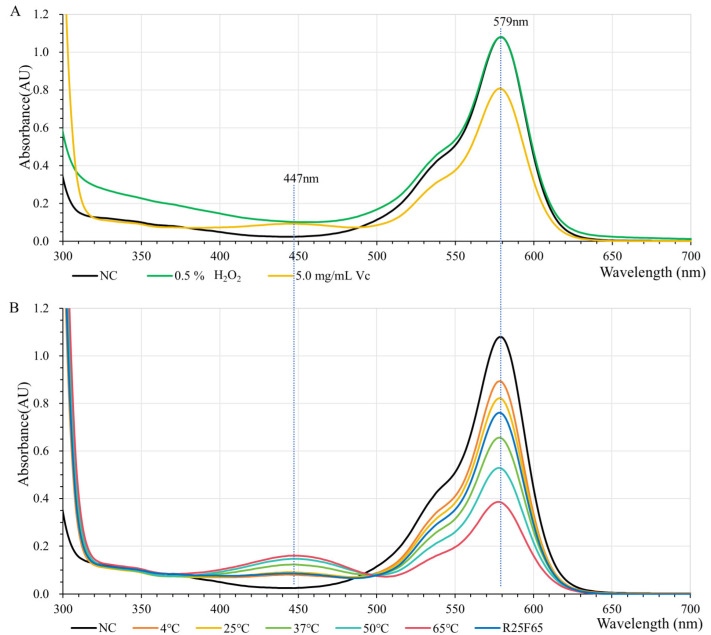
Absorption spectroscopies of GfasPurple treated with H_2_O_2_ or Vitamin C. (**A**) GfasPurple treated with 0.5% H_2_O_2_ or 5.0 mg/mL vitamin C at 37 °C for 60 min. (**B**) Reversible and temperature-related effects of 5.0 mg/mL vitamin C on GfasPurple. R25F65: recovering from 65 °C to 25 °C.

H_2_O_2_ and vitamin C are common oxidants and reducing agents in living organisms that affect the color of chromoproteins. Our test here shows that AeBlue is very sensitive to H_2_O_2_, while sfGFP and mKate2 are sensitive to vitamin C (Figure 3 and Figure S1). GfasPurple is not sensitive to either H_2_O_2_ or vitamin C. Neither of them affects the wavelength of the absorption peak (Figure 5A). The impact of a high concentration of vitamin C on GfasPurple is reversible and temperature-related (Figure 5B). A higher temperature is beneficial for the reaction between vitamin C and GfasPurple, forming more product with greater light absorption ability at around 447 nm as absorption at around 579 nm falls.

### 2.4. The Effect of Acidity and Alkalinity on the Color of GfasPurple

**Figure 6:**
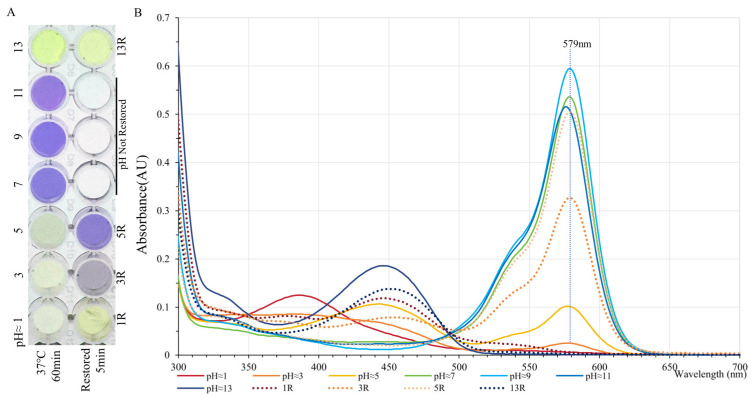
pH tolerance tests of GfasPurple. (**A**) The color of GfasPurple around a given pH in a microplate. (**B**) Corresponding absorption spectroscopies as shown in (**A**) 1R: pH restored from 1 to 9; 3R: pH restored from 3 to 9; 5R: pH restored from 5 to 9; 13R: pH restored from 13 to 9.

Acidity and alkalinity have magical effects on the colors of natural pigments. Chromophores in most chromoproteins are pH-sensitive more often than not. GfasPurple displays purple at pH seven to eleven for a long time, with the strongest absorption at pH around nine, and upon a red shift in absorption peak at pH around eleven, it turns slightly red (Figure 6A,B). Both acidic and alkaline conditions can cause GfasPurple to turn yellow upon blue light absorption (Figure 6B). When pH was adjusted to around five, a recovery with minor loss can be seen, while it barely recovered when pH was adjusted to around one or thirteen.

Moreover, a transitory acid treatment of pH around one for 10 s was conducted. GfasPurple completely lost its color upon the addition of acid, with an immediate reappearance of purple color when pH was restored to around 9. These data suggest that GfasPurple could be particularly useful in applications requiring a stable chromophore in slightly basic conditions.

### 2.5. GfasPurple Variants Display Different Absorption Characteristics in Visible Light Spectrum

**Figure 7:**
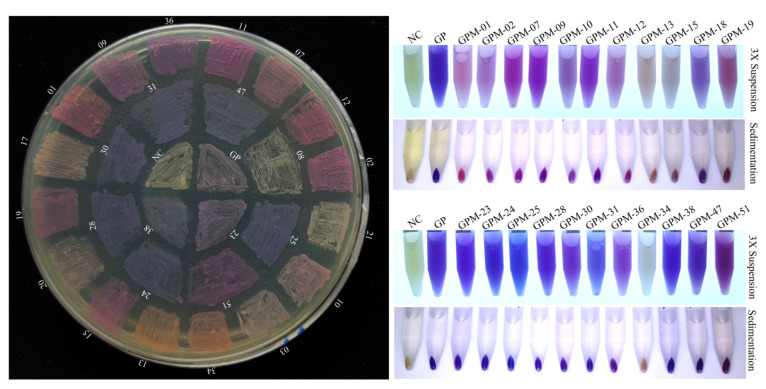
GfasPurple variants display on an LB agar plate, triple enrichment bacterial suspension (3× Suspension in water), and equivalent pellets. GP: GfasPurple; GPM-01 (IC); GMP-02 (LC); GMP-03 (TL); GMP-07 (YC); GMP-08 (VN with E91G); GMP-09 (LT); GMP-10 (SC); GMP-11 (VT); GMP-12 (AS); GMP-13 (IG); GMP-14 (FI); GMP-15 (LS); GMP-18 (VL with A2T); GMP-19 (LL); GMP-21 (SL); GMP-23 (SQ with S219P); GMP-24 (CQ); GMP-25 (VQ); GMP-28 (FQ); GMP-30 (FM); GMP-31 (VM); GMP-34(FN); GMP-36 (VL); GMP-38 (CM); GMP-47 (VN); GMP-51 (FL); the letters in round brackets represent the mutated amino acids at S62 and G63.

**Figure 8:**
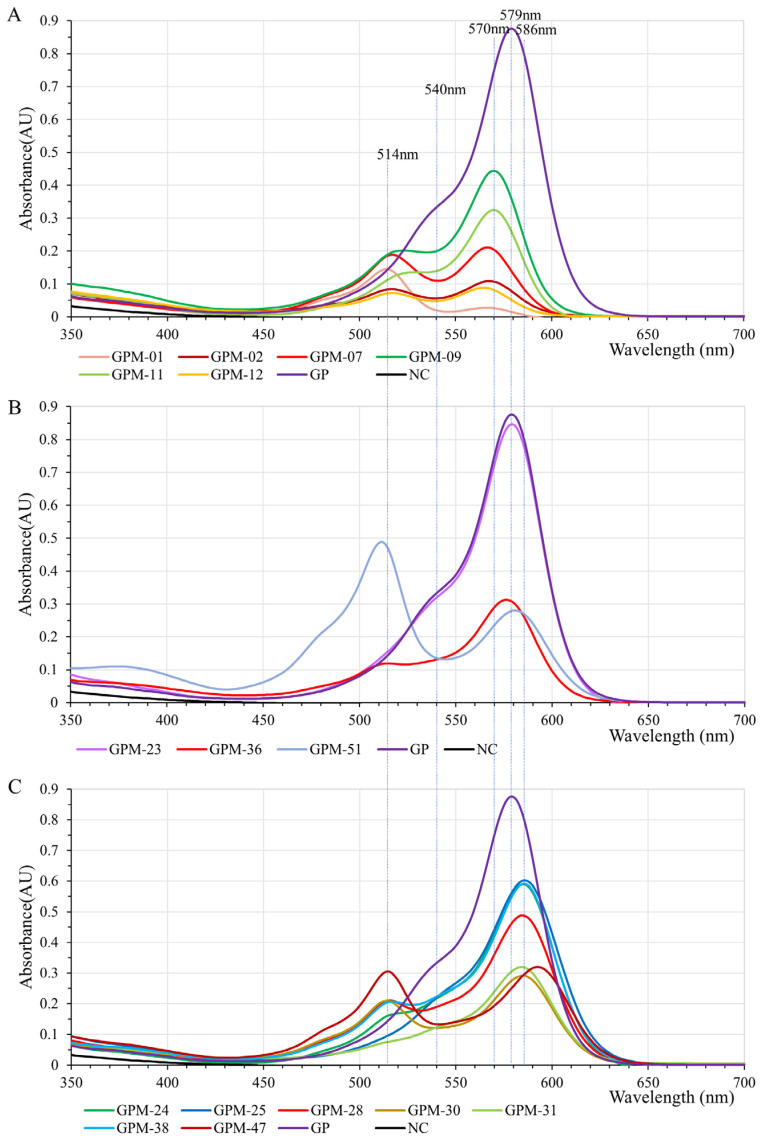
Absorption spectroscopies of GfasPurple variants as shown in Figure 7. (**A**–**C**) correspond to the purplish-red, purple, and bluish-violet variants, respectively.

The chromophore of GfasPurple is highly relevant with S62, G63, Y64, and G65, as described by [8]. The variants acquired via random mutation at S62 and G63 display different colors such as red, purplish red, purple, bluish violet, etc. (Figure 7). Subsequent absorption spectroscopy analyses revealed the basses when they have different colors. Most of the red variants have a red shift from 579 nm to about 570 nm. More than that, they share analogous and clearer vice peaks at around 514nm. In some variants, such as GPM-07 (S62Y and G63C) and GPM-01 (S62I and G63C), this vice peak is equal to or even superior to the typical peak (Figure 8A). Three purple variants are worth paying attention to. GPM-07 has a S219P-directed mutation which makes it more approaching to AmilCP, and it has a limited influence on its absorption spectrum. GPM-51 (S62F and G63L) is a purplish-red variant that has a main peak at 512 nm and a vice peak at 580 nm. GPM-36 (S62V and G63L) is a purple variant with a small red shift to 576 nm and a vice peak at 514 nm (Figure 8B). GPM-25 (S62V and G63Q) is the bluest variant with a blue shift at 586 nm compared to the initial version (Figure 8C), specifically it has no vice peak at around 514 nm. Although GPM-47 (S62V and G63N) has the most obvious blue shift at 592 nm, the vice peak at 514 nm prevents it from turning blue.

## 3. Discussion

Our strategy of directly expressing POCPs in the clone strain using a high-copy-number plasmid is essentially the most cost-effective and concise approach with a single constant condition. All POCPs were well expressed except tdTomato, in which a significant decrease in growth rate was observed when expressed in *E. coli*. There are few reports on the adverse effects of tdTomato on living organisms [18,19].

Due to the lack of the enzymatic amplification effect, the accumulation of primary metabolites is not the preferred route for organisms to become colored [12,20]. Here, GfasPurple is a highly colored POCP with a high molar extinction coefficient at 205,200 M^−1^ cm^−1^; it ranks in the top three in FPbase [6]. The molar extinction coefficient of Aeblue is 110,000 M^−1^ cm^−1^, and this indicates that the high absorption peak of GfasPurple in Figure 2 is mostly due to its high extinction ability rather than protein concentration. In contrast, AmilCP has a higher molar extinction coefficient than the other fluorescent proteins tested here, and the low absorption peak is related to its longer maturity time [17,21].

There is little research on non-fluorescent chromoproteins, and many of them are the by-products of fluorescent protein research [10,11,22,23]. However, these non-fluorescent chromoproteins possess unique properties that make them potential valuable in various applications [24,25]. Yet, there are very few studies on GfasPurple in the published papers relevant to chromoproteins.

Here, GfasPurple exhibited a remarkable tolerance to heat, maintaining its color and functionality even after exposure to temperatures of up to 70 °C. However, when exposed to 50% ethanol, GfasPurple loses its color rapidly at 65 °C (Figure 4). There are two possible explanations for this: GfasPurple either becomes insoluble and precipitates or it is destroyed by ethanol. To distinguish between these two possibilities, supplementary tests were conducted at higher temperatures and with different concentrations of ethanol. GfasPurple has good solubility in 50% ethanol and poor solubility in 80% ethanol. It fades completely at 60 °C in 50% ethanol but keeps its color at higher temperatures in 80% ethanol in the form of an insoluble precipitate (Figure S2B). All these results prove that soluble GfasPurple can be destroyed by ethanol, but insoluble GfasPurple is tougher in high-concentration ethanol.

Furthermore, GfasPurple demonstrated a strong resistance to oxidative stress, as evidenced by its ability to withstand high concentrations of H_2_O_2_ without a significant loss of color. In contrast, the presence of vitamin C led to a noticeable decrease in the absorption peak, indicating that the protein’s chromophore is sensitive to reducing agents. This robustness is a significant advantage for applications where the protein might encounter harsh environmental conditions.

The mutagenesis and screening process for GfasPurple is instrumental in achieving its exceptional properties. This iterative process of mutation and selection led to the development of GfasPurple, a protein that not only has a high molar extinction coefficient but also demonstrates remarkable stability under various environmental conditions. These can provide insights into their potential applications as biosensors, biocolorants, or even synthetic biological targets aimed at food colorants like c-phycocyanin. Based on these findings, GfasPurple may become a protein target for large-scale biosynthesis and low-cost extraction in synthetic biology. Unfortunately, the kinetic rates of GfasPurple degradation and the crystal structures of its variants have not been described, as reported by [21,26].

As for AeBlue, its color varies during the cultivation period at invariant 37 °C. Firstly, it turns red, then purple, and finally stabilizes to blue. This phenomenon is similar to the result previously reported, which was tested at 4 °C [8,16]. During heat tolerance tests, a different color shift was noticed. After heat treatment at 50 °C or 60 °C, AeBlue undergoes denaturation and precipitation. The precipitate’s color shifts from light green to yellow, and it can be excited by blue light to produce green fluorescence, which was not present before heat treatment (Figure S2A). According to previous research, AeBlue can be modified into a far-red fluorescent protein with several mutations [27]. However, the color of matured AeBlue is relatively stable during color fading in a relatively low-temperature environment such as 37 °C. All these results indicate that the chromophore of AeBlue is changing during the maturity process, and it is variable under higher temperature conditions.

## 4. Materials and Methods

### 4.1. Plasmid Construction

A high-copy-number ampicillin-resistant plasmid vector, pSG-CBSA001, was previously constructed for prokaryotic expression, which has a synthetic bacterial EM7 promoter along with the clone sites placed between the phage T3 early transcription terminator and the bidirectional *E. coli* tonB-P14 transcription terminator. The eGFP sequence was amplified from pIRES-eGFP and placed into the *Eco*RI and *Pst*I sites that reside in pSG-CBSA001 via a restriction–ligase reaction. The sequences of sfGFP, mKate2, AmilCP, AeBlue, and GfasPurple were obtained from FPbase “https://www.fpbase.org/ (accessed on 1 May 2024)” with the following FPbase accession IDs: B4SOW, DBBO8, 18KQQ, LFOF4, and OXU4P. These sequences were all codon optimized, giving attention to both eukaryotes and prokaryotes. Then, they were all synthesized using SynbioB or Tsingke and subsequently placed into pSG-CBSA001 to construct the corresponding expression vectors. The DH5α strain harbored relevant plasmids that were used for the dual purpose of cloning and expressing of the host bacteria.

### 4.2. Protein Expression and Extraction

The identified positive bacteria were cultivated in 100 mL LB media supplemented with 75 mg/L ampicillin at 37 °C and 220 rpm for 3 days. The cultivation containers consist of sterile 250 mL conical flasks with a breathable sealing film. Usually, the color of the bacterial cells appears on day 1 and remains stable on day 3 or thereafter, except for sfGFP, which could be stable on day 2. A strain harboring pSG-CBSA001 was synchronously cultivated as the negative control.

Cultivated bacterial cells including the negative control were collected and centrifugated in centrifuge tubes (5000× *g* for 3 min at ambient temperature). Then, they were resuspended in sterile water. The bacterial suspension was lysed using 1 mg/mL egg white lysozyme (Biohonor) at 37 °C for 60 min, followed by a short, violent oscillation and centrifugation at 15,000× *g* for 10 min to remove the cell debris. The supernatants were taken and filtered through a 0.45 μm membrane to obtain clear, crude protein extraction solutions. The extraction solutions were split and stored at −80 °C for later use.

### 4.3. Absorption Spectroscopy Analysis

Clear chromoprotein extraction solutions were tested to obtain UV–visible spectra utilizing a UV-1900i spectrophotometer (Shimadzu, Suzhou, China), which could fully cover the visible light spectrum (normally 750–270 nm, step = 2 nm, room temperature). For samples in 96-well microplates, the absorption analysis was conducted with a SpectraMax iD3 microplate absorbance reader (Molecular Devices, Shanghai, China) at the characteristic absorption wavelength at room temperature.

### 4.4. Physicochemical Tests

#### 4.4.1. Heat and Ethanol Tolerance Tests

Bacteria cultured for 3 days were collected and triple-enrichened; bacterial suspensions were resuspended with water, and equivalent bacterial pellets were treated with water, 75% ethanol, or 95% ethanol at 65 °C for 5 min. Then, their colors were observed and photographed under white and blue excitation light at around 450nm respectively.

The extraction solutions of AeBlue, GfasPurple, mKate2, and sfGFP together with the chromoprotein-less negative control in a 200 μL centrifuge tube were heat treated at 40, 50, 60, 70, 80, and 90 °C for 60 min, followed by centrifugal treatment at 10,000× *g* for 5 min. The supernatants were then transferred into a microplate followed by observation and photographing under white and blue excitation light at around 450 nm, respectively. A camera equipped with an additional blue light filter baffle is necessary when exposed to an excitation light source. Untreated samples temporarily stored at 4 °C were used as the contrast.

The extraction solutions of GfasPurple in a 2 mL centrifuge tube were heat treated in water or with 56% or 75% ethanol at 65 °C for 5, 10, 20, 30, 60, and 120 min synchronously, followed by centrifugal treatment at 10,000× *g* for 5 min. Then, parts of the supernatants were transferred into a microplate followed by observation and photographing under white light. The rest of the supernatant was used as a sample in the absorption spectroscopy analysis just shown above.

#### 4.4.2. H_2_O_2_ and Vitamin C Tolerance Tests

The extraction solutions of AeBlue, GfasPurple, mKate2, and sfGFP together with the chromoprotein-less negative control in a 200 μL centrifuge tube were treated with 0.5%, 0.1%, and 0.02% H_2_O_2_ or with 5.0 mg/mL, 1.0 mg/mL, and 0.2 mg/mL vitamin C for 60 min at 37 °C. Then, the reaction solutions were transferred into a microplate followed by observation and photographing under white and blue excitation light at around 450 nm, respectively. Samples with no H_2_O_2_ or vitamin C were synchronously processed and used as the contrast.

The extraction solutions of GfaPurple in a 2 mL centrifuge tube were treated with 5.0 mg/mL vitamin C at 37 °C for 60 min, and then the absorption spectroscopy analysis was performed at 4, 25, 37, 50, and 65 °C, finally finishing at 25 °C in the same cuvette. Before each test, samples together with the cuvette were balanced for 5 min at the corresponding temperature.

#### 4.4.3. pH Tests

The pH of the GfaPurple extraction solutions in a 5 mL centrifuge tube were adjusted to about 1, 3, 5, 7, 9, 11, and 13, followed by an incubation at 37 °C for 60 min. Thereafter, some of the solution was readjusted to about 9. Then, the solutions were partly transferred into a microplate followed by observation and photographing under white light. The remaining samples were used for absorption spectroscopy detection.

### 4.5. GfasPurple Mutagenesis and Screening

Site-directed mutagenesis was performed via fusion polymerase chain reaction (PCR) with the KOD Neo Plus polymerase (TOYOBO). The upstream primer 5′-TACGGATCTATACCATTCACCAAG-5-3′ and a downstream primer 5′-GGTGAATGGTATAGATCCGTANNNNNN-CTGTGGTGAAAGAATATCCCAG-3′ were used to create the mutation sequences of GfasPurple. These mutation sequences were placed into pSG-CBSA001 via the restriction–ligase reaction. The reaction products were then transformed into the competent cell of DH5α, followed by cultivation at 37 °C for 48 h. Interesting bacterial colonies with conspicuous colors were picked, stored, and subsequently verified via Sanger sequencing. Repeated transformants were removed before subsequent tests.

## 5. Conclusions

Here, a high-copy-number plasmid vector has been proven to work well in the prokaryotic expression of POCPs at very low cost. GfasPurple is the best biocolorant we have ever tested, and it has better stability against heat, acid, alkali, high-concentration ethanol, H_2_O_2_, or vitamin C. The GfasPurple mutagenesis and screening process led to the identification of several mutant variants with altered absorption spectra. These mutants could be valuable for further studies aimed at understanding the structure–function relationship of the protein and for employing its properties in specific applications, such as biosensors or bioimaging agents. Even though we have not obtained a pure-blue variant of GfasPurple as desired, this result has given us a better direction to search for target variants with various colors based on a relatively stable POCP.

## Data Availability

The original contributions presented in the study are included in the article/Supplementary Materials. Further inquiries can be directed to the corresponding author.

## Supplementary Materials

The following supporting information can be downloaded at the following website: https://www.mdpi.com/article/10.3390/molecules30030432/s1, Figure S1: Light absorption associated with Figure 3 at the characteristic absorption wavelength; Figure S2: Supplementary diagrams in thermal stability tests; Sequence information: Amino acid sequences of protein-only chromoproteins used in this study.
