# Supplementary material for: Colored Proteins Act as Biocolorants in Escherichia coli
Source: Molecules. 2025 Jan 21;30(3):432. doi: 10.3390/molecules30030432 (PMC11819954; doi:10.3390/molecules30030432)
Supplement: Supplementary file 1 [file molecules-30-00432-s001.zip › Sequence information.pdf]

Amino Acid Sequences of Protein-only Chromoproteins Used in This Study.

1. GfasPurple:
   ASVIAKQMTYKVYMSGTVNGHYFEVEGDGKGKPYEGEQTVKLTVTKGGPLPFA
   WDILSPQSQYGSIPFTKYPEDIPDYVKQSFPEGYTWERIMNFEDGAVCTVSNDSSI
   QGNCFIYHVKFSGLNFPPNGPVMQKKTQGWEPNTERLFARDGMLIGNNFMALKL
   EGGGHYLCEFKSTYKAKKPVKMPGYHYVDRKLDVTNHNKDYTSVEQCEISIAR
   KSVVA*
2. AmilCP:
   MSVIAKQMTYKVYMSGTVNGHYFEVEGDGKGKPYEGEQTVKLTVTKGGPLPFA
   WDILSPQCQYGSIPFTKYPEDIPDYVKQSFPEGYTWERIMNFEDGAVCTVSNDSSI
   QGNCFIYHVKFSGLNFPPNGPVMQKKTQGWEPNTERLFARDGMLLGNNFMALK
   LEGGGHYLCEFKTTYKAKKPVKMPGYHYVDRKLDVTNHNKDYTSVEQCEISIAR
   KPVVA*
3. AeBlue:
   MASLVKKDMCIKMTMEGTVNGHHFKCVGEGEGKPFEGTQVEKIRITEGGPLPFA
   YDILAPCCMYGSKTFIKHVSGIPDYFKESFPEGFTWERTQIFEDGGYLTIHQDTSLQ
   GNNFIFKVNVIGANFPANGPVMQKKTAGWEPCVEMLYPRDGVLCGQSLMALKC
   TDGNHLTSHLRTTYRSRKPSNAVNMPEFHFGDHRIEILKAEQGKFYEQYESAVAR
   YCEAAPSKLGHH*
4. eGFP:
   MVSKGEELFTGVVPILVELDGDVNGHKFSVSGEGEGDATYGKLTLKFICTTGKLP
   VPWPTLVTTLTYGVQCFSRYPDHMKQHDFFKSAMPEGYVQERTIFFKDDGNYKT
   RAEVKFEGDTLVNRIELKGIDFKEDGNILGHKLEYNYNSHNVYIMADKQKNGIK
   VNFKIRHNIEDGSVQLADHYQQNTPIGDGPVLLPDNHYLSTQSALSKDPNEKRDH
   MVLLEFVTAAGITLGMDELYK*
5. sfGFP:
   MVSKGEELFTGVVPILVELDGDVNGHKFSVRGEGEGDATNGKLTLKFICTTGKLP
   VPWPTLVTTLTYGVQCFSRYPDHMKRHDFFKSAMPEGYVQERTISFKDDGTYKT
   RAEVKFEGDTLVNRIELKGIDFKEDGNILGHKLEYNFNSHNVYITADKQKNGIKA
   NFKIRHNVEDGSVQLADHYQQNTPIGDGPVLLPDNHYLSTQSVLSKDPNEKRDH
   MVLLEFVTAAGITHGMDELYK*
6. mKate2:
   MVSELIKENMHMKLYMEGTVNNHHFKCTSEGEGKPYEGTQTMRIKAVEGGPLPF
   AFDILATSFMYGSKTFINHTQGIPDFFKQSFPEGFTWERVTTYEDGGVLTATQDTSL
   QDGCLIYNVKIRGVNFPSNGPVMQKKTLGWEASTETLYPADGGLEGRADMALKL

VGGGHLICNLKTTYRSKKPAKNLKMPGVYYVDRRLERIKEADKETYVEQHEVAVARYCDLPSKLGHR*

7. tdTomato

MVSKGEEVIKEFMRFKVRMEGSMNGHEFEIEGEGEGRPYEGTQTAKLKVTKGGPLPFAWDILSPQFMYGSKAYVKHPADIPDYKKLSFPEGFKWERVMNFEDGGLVTVTQDSSLQDGTLIYKVKMRGTNFPPDGPVMQKKTMGWEASTERLYPRDGVLKGEIHQALKLKDGGHYLVEFKTIYMAKKPVQLPGYYYVDTKLDITSHNEDYTIVEQYERSEGRHHLFLGHGTGSTGSGSSGTASSEDNNMAVIKEFMRFKVRMEGSMNGHEFEIEGEGEGRPYEGTQTAKLKVTKGGPLPFAWDILSPQFMYGSKAYVKHPADIPDYKKLSFPEGFKWERVMNFEDGGLVTVTQDSSLQDGTLIYKVKMRGTNFPPDGPVMQKKTMGWEASTERLYPRDGVLKGEIHQALKLKDGGHYLVEFKTIYMAKKPVQLPGYYYVDTKLDITSHNEDYTIVEQYERSEGRHHLFLYGMDELYK*
