# Supplementary material for: Colored Proteins Act as Biocolorants in Escherichia coli
Source: Molecules. 2025 Jan 21;30(3):432. doi: 10.3390/molecules30030432 (PMC11819954; doi:10.3390/molecules30030432)
Supplement: Supplementary file 1 [file molecules-30-00432-s001.zip › Supplementary Figures.pdf]

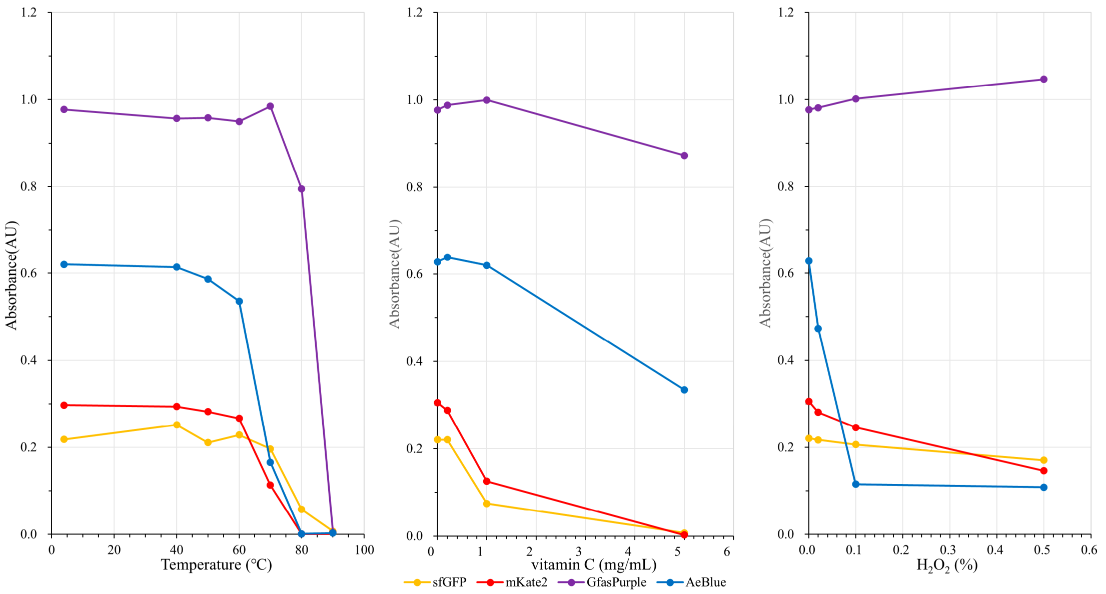

Figure S1. Light absorption associated with Fig.3 at characteristic absorption wavelength. sfGFP, mKate2, AeBlue, GfasPurple at 488nm, 588nm, 596nm, 578nm correspondingly.

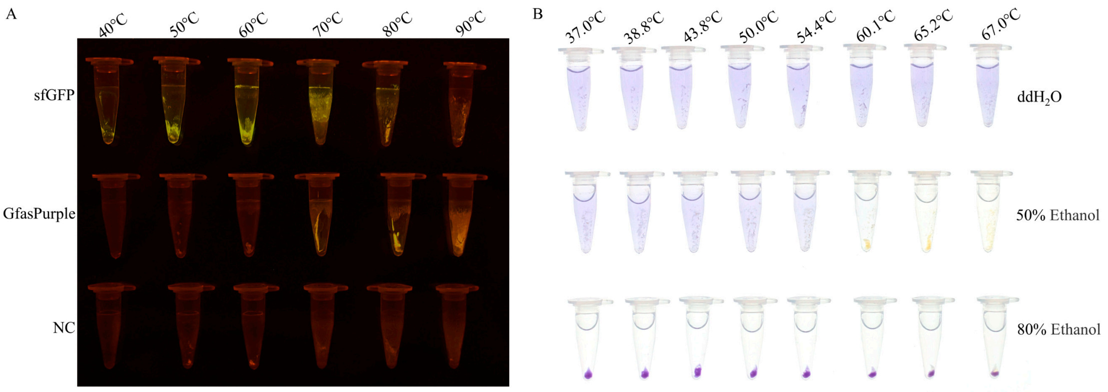

Figure S2. Supplementary diagrams in thermal stability tests. (A) Fluorescence observation of precipitation caused by heat treatment associated with Fig.3 under blue excitation light. (B) Supplementary thermal stability tests of GfasPurple at different ethanol concentrations and temperatures for 60 minutes. Samples are centrifuged at 10000g for 5 minutes before taking photos.
